# Acute Retinal Necrosis versus Progressive Outer Retinal Necrosis

**DOI:** 10.1007/s40135-026-00351-2

**Published:** 2026-03-31

**Authors:** Kisha Kalra, Piero Carletti, Raquel Goldhardt

**Affiliations:** 1 Department of Ophthalmology, Bascom Palmer Eye Institute, University of Miami Miller School of Medicine, 900 NW 17th Street, Miami, FL 33136, USA

**Keywords:** Acute retinal necrosis, Progressive outer retinal necrosis, Herpetic viral retinopathy, Viral retinopathies, Varicella zoster virus, Immuno compromised host, Combined antiviral therapy

## Abstract

**Purpose of Review:**

This review aims to provide a comprehensive comparison between acute retinal necrosis (ARN) and progressive outer retinal necrosis (PORN), two sight-threatening manifestations of herpetic viral retinopathies emphasizing how immune status shapes presentation, diagnosis, management, and prognosis.

**Recent Findings:**

Recent studies suggest that ARN and PORN represent a spectrum influenced largely by host immune status rather than distinct diseases. Ultra-widefield imaging improves detection of far-peripheral lesions, while PCR of aqueous or vitreous enables rapid etiologic confirmation. In ARN, high-bioavailability oral antivirals, often with adjunct intravitreal injections, accelerate lesion regression and reduce fellow-eye involvement. In PORN, monotherapy performs poorly; combined intensive intravitreal and systemic therapy improves local control but vision remains limited in advanced immunosuppression.

**Summary:**

Prompt differentiation based on inflammatory signs, lesion morphology, and host immunity directs therapy. ARN generally responds to systemic agents yet carries substantial detachment risk; PORN progresses quickly with worse outcomes despite aggressive care. Early diagnosis coordinated medical–surgical management, and close surveillance are critical; priorities include optimal dosing and duration of antiviral therapies as well as adjunctive anti-inflammatory strategies.

## Introduction

Acute retinal necrosis (ARN) and prgressive outer retinal necrosis (PORN) are both types of herpetic retinopathies, which are considered ophthalmic emergencies due to their rapid progression. Emerging evidence suggests that ARN and PORN represent two poles on a disease continuum of herpetic necrotizing retinopathies rather than completely distinct entities. Understanding both their similarities and differences is crucial for diagnosis, treatment, and patient prognosis.

Although ARN and PORN share significant overlap, several key differences set them apart. While the most common etiology for both of these entities is the varicella zoster virus (VZV), ARN typically presents unilaterally with considerable ocular inflammation in immunocompetent individuals [1]. In contrast, PORN most often affects immunocompromised patients, typically presents with minimal or no inflammation, and is more likely to be bilateral, rapidly progressive, and associated with a worse prognosis than ARN. This review summarizes the key epidemiologic, clinical, and therapeutic aspects of ARN and PORN, emphasizing their overlap and distinctive features relevant for clinical management.

## Background and Etiology

### ARN

Urayama et al. initially described the entity in 1971 as ‘Kirisawa’s Uveitis,’ and subsequent studies using electron microscopy identified VZV as the causative agent. ARN is most frequently caused by VZV, followed by Herpes Simplex Virus (HSV), Cytomegalovirus (CMV), and Epstein-Barr Virus (EBV) [2]. Understanding the viral spectrum is important since therapeutic response and recurrence risk may vary by viral subtype.

### PORN

This etiologic overlap further supports the theory that both disorders belong to a unified herpetic retinal necrosis spectrum, modulated by host immune status.

## Epidemiology

### ARN

ARN typically affects immunocompetent individuals but can also occur in those with transient immunosuppression (e.g., due to medications). Older adults are more commonly affected, likely due to waning immunity to VZV, while younger patients, particularly those under twenty-five, are more likely to have HSV-2.[4, 5] Gender predilection has not been consistently demonstrated across studies, though some studies show a higher incidence in males. ARN can occur several years after the primary infection, and several genetic associations have been noted, such as HLA-Aw33, HLA-B44, and HLA-DRw6 in the Japanese population, and HLA-DQw7, HLA-Bw62, and HLA-DR4 in White patients [6].

### PORN

PORN is primarily seen in severely immunocompromised patients, including those with HIV or Acquired Immuno-deficiency Syndrome (AIDS), those undergoing immune-suppressive therapies (e.g., solid organ or bone marrow transplants, chemotherapy, or for the treatment of rheumatologic conditions), and patients with leukemic processes [7–10]. In HIV patients, the incidence of PORN increases significantly when the CD4 + count falls below 50. There is limited epidemiological data for PORN, but in patients with AIDS, the median age is thirty-seven, suggesting that it affects a younger population compared to ARN [8].

In ARN, most affected patients are immunocompetent older adults, whereas PORN primarily affects immune-compromised individuals with advanced HIV or iatrogenic immunosuppression.

## Pathophysiology

### ARN

In ARN, the virus infiltrates healthy retinal tissue, triggering an acute inflammatory response characterized by lymphocyte and plasma cell migration, which often leads to vitritis. The viral infiltration also causes retinal arterial occlusions and widespread retinal vasculitis and necrosis [2, 4, 11] Vitreous inflammation can also result in contractile membranes that can lead to retinal detachments, which typically occur within three months of onset. The type of virus has not been shown to affect the rates of retinal detachment [12, 13].

### PORN

The pathophysiology of PORN mirrors that of ARN in that viral infiltration of retinal cells occurs. However, histologic studies suggest that PORN involves full-thickness retinal damage rather than just outer retinal layers [14–16].

In contrast to ARN, PORN shows much less intraocular inflammation and a less prominent vasculitic component, likely due to the immunocompromised status of the patients. Less than 40% of PORN cases present with anterior chamber reaction, and less than 20% show vitreous inflammation, which is in stark contrast to the significant intraocular inflammation seen in ARN [8, 17].

Thus, while both entities involve viral-induced retinal necrosis, the host’s immune status largely determines the extent of inflammation and vascular involvement.

## Histology and Cytopathology

Histologic analysis further highlights the pathophysiologic distinctions between the two. These findings suggest that the immune privilege of the eye is partially overridden during active infection.

### ARN

Histologically, necrotic retinal cells in ARN exhibit eosinophilic intranuclear inclusions. Electron microscopy reveals viral infiltration across all retinal layers [18]. Cytopathological analysis of vitreous samples from eyes with ARN undergoing pars plana vitrectomy (PPV) shows significant involvement of both the innate and adaptive immune response. In one study of 14 eyes, 71% tested positive for HSV and 29% for VZV. Most eyes displayed lymphocyte proliferation, macrophages or histiocytes, and polymorphonuclear cells [19].

### PORN

Histologic findings in early PORN, before full retinal involvement, are less well documented. It was long believed that necrosis starts in the outer retina, but newer reports indicate that inner retinal layers can also be involved early on.[20]. Advanced disease leads to full-thickness necrosis and architectural collapse of the retina, correlating with rapid and irreversible vision loss. The inflammatory infiltrates are predominantly lymphocytic, with few macrophages. Lymphocytic infiltration is also observed in the choroid and choriocapillaris [15, 20].

## Clinical Presentation and Symptoms

ARN typically presents with ocular pain and pronounced inflammation, while PORN progresses silently with minimal symptoms. A clinical pearl when examining a patient with possible viral retinitis is that funduscopic examination in ARN reveals perivascular sheathing and patchy necrosis, whereas PORN often manifests with confluent, creamy-white lesions resembling ‘cracked mud.’ See Table 1 for key differentiating features.

### ARN

Patients with ARN typically present with rapid loss of vision, ocular or periocular pain, redness, floaters, and photophobia. Typical age of presentation is around 50 years old, though it can affect younger patients as well. Retinal lesions often develop about a week after the onset of symptoms. While most cases are unilateral, up to 30% can become bilateral. Patients should be asked about any history of immunosuppression, HIV/AIDS, prior ocular treatments, surgeries, or systemic conditions. A comprehensive exam, including a dilated fundus exam with scleral depression, is essential for detecting retinal whitening, vascular sheathing, or hemorrhages [21–23].

### PORN

In contrast to ARN, PORN generally presents with minimal intraocular inflammation. Lesions are typically yellow-white, creamy, and peripheral, which eventually coalesce into larger patches. Notably, patients are less likely to present with ocular pain compared to ARN, likely due to a reduced inflammatory response. PORN is more likely to affect both eyes and progress more rapidly. Many patients also show signs of cutaneous VZV infection (shingles) or CNS involvement like meningitis or vasculitis [3, 8].

## Diagnosis

**Fig. 1:**
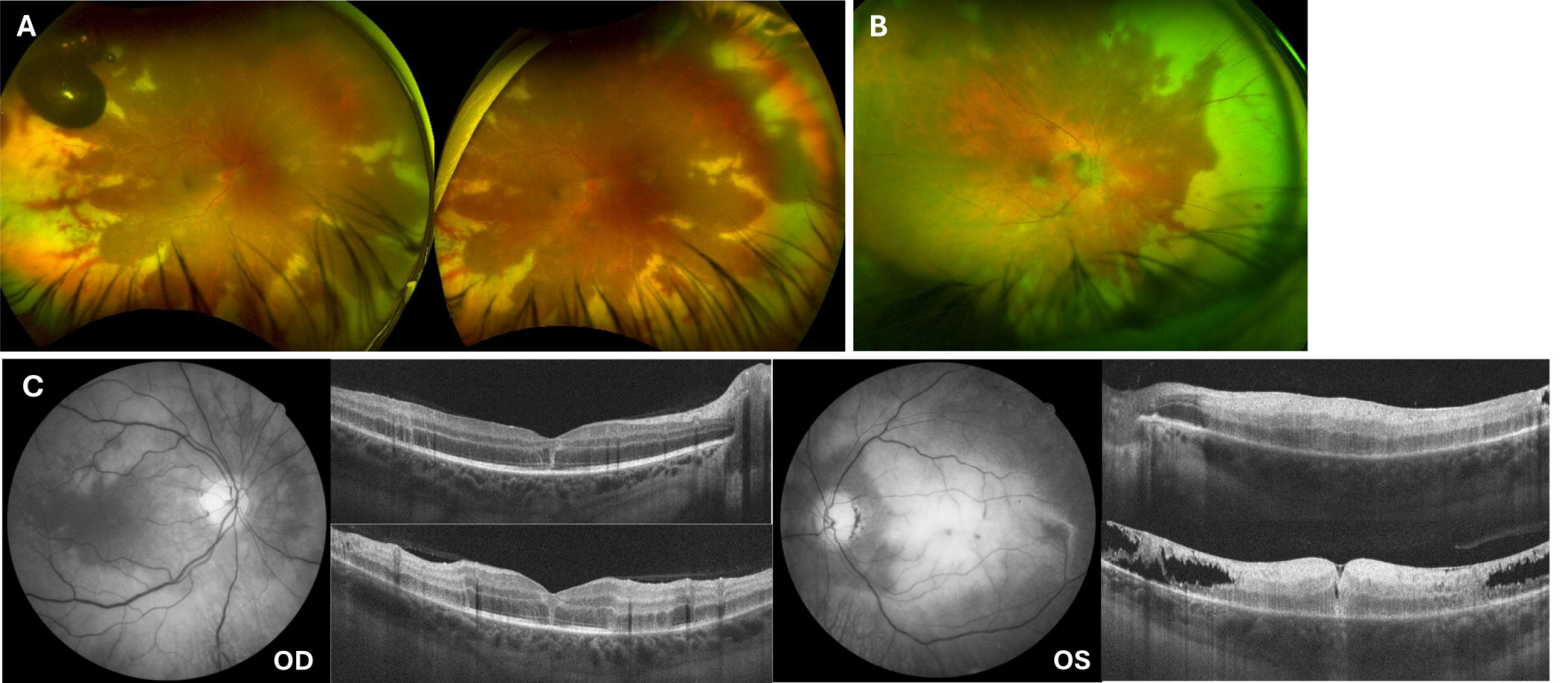
(A) Color fundus photograph of the right eye of a 28-year-old male with PCR-confirmed VZV-ARN, showing peripheral retinal whitening and necrosis. (B) Color fundus photograph of the right eye of a 78-year-old female with PCR-confirmed VZV-ARN, demonstrating confluent retinal necrosis and vasculitis. (C) Red-free fundus photographs of the right and left eyes of a 41-year-old immunosuppressed male (bone marrow aplasia after bone marrow transplant, on prednisone) with PCR-confirmed VZV-PORN, illustrating bilateral multifocal outer retinal necrosis. Optical coherence tomography (OCT) in this PORN case reveals: Right eye (OD) – severe outer retinal destruction with near-complete loss of the ellipsoid zone and RPE, marked retinal thinning, and hyper-reflective outer retinal and subretinal deposits. Left eye (OS) – diffuse outer retinal atrophy with patchy photoreceptor/RPE loss and subretinal hyper-reflective material, with partial preservation of the foveal contour. *Photos for C courtesy of Mariana R. Thorell and Roberto P. P. Schunemann*)

Diagnostic confirmation should integrate clinical findings, imaging (UWFI, OCT), and molecular testing (PCR for HSV/VZV). Representative multimodal imaging findings are shown in Fig. 1.

### Differential diagnosis or mimickers

Conditions that can resemble necrotizing herpetic retinopathy, particularly when intraocular inflammation is minimal as in PORN—include cytomegalovirus (CMV) retinitis (often granular necrosis with hemorrhage in advanced immunosuppression), ocular toxoplasmosis (typically focal retinochoroiditis with vitritis), syphilitic posterior uveitis/retinitis (variable vasculitis and multifocal lesions), endogenous endophthalmitis (prominent vitreous involvement and systemic infectious risk factors), and intraocular lymphoma/masquerade syndromes. Correlation with host immune status, lesion morphology (vasculitis and dense vitritis favor ARN), multimodal imaging, and rapid aqueous/ vitreous PCR testing are critical to avoid delays in appropriate therapy.

### ARN

The diagnostic criteria for ARN were first established in 1994 by the American Uveitis Society (AUS) and revised in 2015 by the Japanese ARN Study Group and in 2021 by the Standardization of Uveitis Nomenclature (SUN) Working Group [1, 24, 25]. The most widely used criteria come from the SUN Working Group. These criteria diagnose ARN based on the presence of necrotizing retinitis in the peripheral retina AND evidence of infection with HSV or VZV from aqueous or vitreous specimen or characteristic clinical picture, including rapid progression without antiviral treatment, circumferential spread of the disease, evidence of occlusive vasculopathy, and prominent inflammatory reaction in the vitreous and anterior chamber.

Ultra-wide field fundus imaging (UWFI) has proven useful in diagnosing ARN, particularly for detecting lesions in the far periphery and assessing progression, regression, or recurrence (see Fig. 1A-B) [26] PCR testing for HSV or VZV from vitreous or aqueous samples is also highly sensitive, with a positive rate of 79–100% in ARN cases [5, 23]. However, treatment should not be delayed while waiting for PCR results if clinical suspicion is high.

### PORN

While no formal diagnostic criteria exist for PORN, it is typically diagnosed based on multifocal lesions in the peripheral retina that rapidly coalesce. Fundus exams often reveal a “cracked mud” appearance due to scarring and sclerotic vessels (See Fig. 1C). As with ARN, PCR testing and ophthalmic imaging can assist with diagnosis.

Timely recognition is essential, as therapeutic delay, even by days, can drastically alter visual prognosis.

In any patient with suspected necrotizing herpetic retinopathy, obtain anterior chamber or vitreous fluid for PCR testing and initiate empiric systemic antiviral therapy immediately. Immunocompetent patients with prominent inflammation and occlusive vasculitis are more likely to have ARN, whereas severely immunosuppressed patients with minimal inflammation and rapidly progressive multifocal outer retinal lesions are characteristic of PORN. Adjunctive intravitreal antivirals and close retinal detachment surveillance are recommended in both entities.

## Treatment

### ARN

Systemic antivirals form the cornerstone of ARN management and have been shown to reduce bilateral involvement dramatically. Prior to the availability of intravitreal antivirals, intravenous acyclovir was the primary option. The typical dosing for intravenous acyclovir was 1500 mg/m^2^/ day for 7–10 days, followed by oral acyclovir for an additional two-week period. However, the advent of oral antivirals with high bioavailability, such as valacyclovir, has led to these medications becoming the preferred option, as they are equally effective and provide satisfactory vitreous penetration [27]. A study by Tibbets et al. found that valacyclovir, dosed at 2 g three times a day, offered better visual outcomes compared to the 1-gram three-times-a-day regimen [28]. Systemic antiviral therapy has been shown to induce regression of retinal lesions within as little as two days and also significantly reduces the risk of contralateral eye involvement compared to untreated patients [29]. While valacyclovir is a medication with a favorable adverse effect profile and is generally well tolerated, renal dosing is an important consideration. The dosing is determined based on creatinine clearance; if is equal to or above 50, the dosing is not adjusted; if it is 49 or below, the dose and/or dosing intervals need to be adjusted. In patients on hemodialysis or peritoneal dialysis, the dose needs to be reduced significantly.

Several studies have shown that combining systemic and intravitreal antiviral treatments can yield better visual outcomes and decrease the risk of retinal detachment. In cases that are particularly resistant to treatment, combining intravitreal ganciclovir with foscarnet may offer additional benefits. However, intravitreal therapy alone is not recommended, as it does not protect the unaffected eye [17, 30–32].

The role of prophylactic laser photocoagulation (PRP) and vitrectomy has been explored because a large percentage of these patients develop vision-threatening retinal detachments. Some studies suggest that pars plana vitrectomy (PPV) without a pre-existing detachment may reduce the rate of retinal detachment; however, this strategy remains controversial given the lack of data to support it as well as the theoretical increase in the risk of proliferative vitreoretinopathy (PVR) [13]. The role of PRP is also controversial and may not offer significant benefit in preventing rates of retinal detachment [22, 33].

### PORN

In contrast to ARN, due to the profound immunosuppression in these patients, monotherapy with either local or systemic antivirals is often inadequate; combination systemic and intravitreal antiviral therapy is preferred. The main treatment approach is a combination of intravitreal antivirals (foscarnet +/− ganciclovir) and systemic therapy with high-dose valacyclovir or intravenous acyclovir. Intravitreal antivirals may be administered up to twice a week during the induction phase, after which the frequency can be tapered based on clinical response. This combined therapy has shown to improve local disease control and may help delay disease progression [17].

One significant study assessing 67 eyes with PORN in HIV patients revealed that early response to intravitreal ganciclovir injections may correlate with better visual outcomes, although the results did not reach significance levels. Those who did not exhibit clear early improvement to intravitreal therapy showed significantly poorer visual outcomes. Additionally, patients who were on HAART (Highly Active Antiretroviral Therapy) at the time of presentation had a reduced rate of retinal detachment compared to those who were not [17].

## Prognosis

### ARN

The prognosis for ARN without prompt antiviral therapy is poor, with rapid progression to blindness in the affected eye within days. If untreated, ARN can lead to bilateral involvement in up to 70% of cases. However, with timely initiation of high-dose systemic antivirals, the risk of bilateral disease can be reduced to about 13%, and the progression of retinal necrosis can be halted [29]. Unfortunately, despite treatment, visual outcomes remain guarded due to irreversible retinal damage and complications such as retinal detachment.

Approximately 50% of eyes with ARN will develop a retinal detachment over the course of the disease,^30^ with some studies suggesting a higher risk in cases with greater retinal involvement (greater than 25% of retina), younger age at onset, and VZV as the underlying viral etiology [21].

Despite aggressive management, ARN remains one of the most devastating viral retinopathies.

### PORN

The prognosis for PORN is generally worse than that of ARN. Most patients develop severe, permanent, and often bilateral vision impairment. Without treatment, about 67% of cases will result in blindness in the affected eye, and 61% will involve the other eye. Even with the advent of intravitreal antiviral therapies, only a small percentage of eyes recover useful vision. In one large series, most eyes with PORN did not achieve visual acuity better than counting fingers. Early response to treatment may marginally improve outcomes, but the overall prognosis remains poor. Factors such as retinal detachment, macular involvement at presentation, and delayed initiation of therapy are strongly associated with worse outcomes [8, 17].

While both conditions carry significant morbidity, early antiviral initiation remains the strongest predictor of visual preservation.

## Conclusion

ARN and PORN represent two ends of the spectrum of necrotizing herpetic retinopathies. ARN generally affects immunocompetent or mildly immunosuppressed adults and is characterized by intense intraocular inflammation and occlusive vasculitis, while PORN occurs almost exclusively in severely immunocompromised patients and demonstrates rapid outer retinal necrosis with little inflammation (see Table 1). Prompt diagnosis using PCR and aggressive antiviral therapy remain essential to preserve vision and prevent retinal detachment.

Future studies focusing on antiviral resistance, adjunctive anti-inflammatory therapy, and genetic susceptibility may further refine management.

Ongoing research and evolving clinical practice in necrotizing herpetic retinitis are focused on improving diagnostic precision, optimizing antiviral strategies, and mitigating vision-threatening complications. Molecular diagnostics, particularly rapid PCR testing of aqueous or vitreous samples, continue to gain prominence for early and specific identification of causative herpesviruses such as VZV, HSV, and CMV, which facilitates targeted therapy and may reduce delays associated with empirical treatment alone. Additional future directions will likely explore the frequency and duration of treatment, use of adjuvant treatments such as corticosteroids, and refining imaging modalities to improve detection and diagnosis [34–36].

## Figures and Tables

**Table 1:** Acute retinal necrosis (ARN) vs. progressive outer retinal necrosis (PORN)

Feature	ARN	Both	PORN
Typical Host	Immunocompetent or mildly immunosuppressed adults	Necrotizing herpetic retinopathy	Severely immunocompromised (AIDS, transplant, high-dose steroids)
Etiology	VZV most common; also HSV	Herpesvirus infection confirmed by PCR	VZV overwhelmingly predominant
Laterality	Usually unilateral at presentation	May become bilateral without treatment	Typically bilateral at presentation
Inflammation	Prominent anterior chamber and vitreous inflammation	Retinal whitening and necrosis	Minimal or absent intraocular inflammation
Vasculitis	Occlusive arteritis and perivascular sheathing	Viral infiltration across all retinal layers	Little or no vasculitis
Symptoms	Pain, redness, photophobia, floaters, rapid vision loss	Progressive visual decline	Often painless with rapid bilateral visual loss
Lesion Pattern	Coalescing peripheral white-yellow lesions	Full-thickness necrotizing retinitis	Multifocal creamy outer retinal lesions (“cracked mud” appearance)
Progression	Slower than PORN (days)	Acute worsening without treatment	Rapid (hours to days)
Histology	Eosinophilic intranuclear inclusions; mixed inflammatory infiltrate	Viral infiltration across all retinal layers	Early outer retinal necrosis progressing to full-thickness collapse
Diagnostic Criteria	Standard American Uveitis Society criteria	PCR testing is essential	No formal criteria; based on host + morphology
Treatment Response	Typically responds to high-dose systemic antivirals ± intravitreal therapy	Requires prompt antiviral initiation	Poor response to monotherapy; requires combined systemic + intravitreal antivirals
Retinal Detachment Risk	High (~ 50% of eyes)	Major vision-threatening complication	Very high; often early and bilateral
Prognosis	Guarded but better than PORN	Vision-threatening disease	Poor; most eyes do not regain functional vision

## Data Availability

No datasets were generated or analysed during the current study.

## References

[1] Holland GN. Standard diagnostic criteria for the acute retinal necrosis syndrome. Executive Committee of the American Uveitis Society. Am J Ophthalmol. 1994;117(5):663–7. 10.1016/s0002-9394(14)70075-3. 8172275

[2] Wong RW, Jumper JM, McDonald HR, Emerging concepts in the management of acute retinal necrosis. Br J Ophthalmol. 2013;97(5):545–52. 10.1136/bjophthalmol-2012-301983. 23235944

[3] Forster DJ, Dugel PU, Frangieh GT, Liggett PE, Rao NA. Rapidly progressive outer retinal necrosis in the acquired immunodeficiency syndrome. Am J Ophthalmol. 1990;110(4):341–8. 10.1016/s0002-9394(14)77012-6. 2220967

[4] Durand ML, Barshak MB, Sobrin L. Eye Infections. N Engl J Med. 2023;389(25):2363–75. 10.1056/NEJMra2216081. 38118024

[5] Ganatra JB, Chandler D, Santos C, Kuppermann B, Margolis TP. Viral causes of the acute retinal necrosis syndrome. Am J Ophthalmol. 2000;129(2):166–72. 10.1016/s0002-9394(99)00316-5. 10682968

[6] Holland GN, Cornell PJ, Park MS, An association between acute retinal necrosis syndrome and HLA-DQw7 and phenotype Bw62, DR4. Am J Ophthalmol. 1989;108(4):370–4. 10.1016/s0002-9394(14)73303-3. 2801857

[7] Chung H, Kim KH, Kim JG, Lee SY, Yoon YH. Retinal complications in patients with solid organ or bone marrow transplantations. Transplantation. 2007;83(6):694–9. 10.1097/01.tp.0000259386.59375.8a. 17414700

[8] Engstrom RE Jr., Holland GN, Margolis TP, The progressive outer retinal necrosis syndrome. A variant of necrotizing herpetic retinopathy in patients with AIDS. Ophthalmology. 1994;101(9):1488–502. 10.1016/s0161-6420(94)31142-0. 8090452

[9] Lewis JM, Nagae Y, Tano Y. Progressive outer retinal necrosis after bone marrow transplantation. Am J Ophthalmol. 1996;122(6):892–5. 10.1016/s0002-9394(14)70391-5. 8956649

[10] Turno-Krçcicka A, Tomczyk-Socha M, Zimny A. Progressive outer retinal necrosis syndrome in the course of systemic lupus erythematosus. Lupus. 2016;25(14):1610–4. 10.1177/0961203316646464. 27178013

[11] Verjans GM, Feron EJ, Dings ME, T cells specific for the triggering virus infiltrate the eye in patients with herpes simplex virus-mediated acute retinal necrosis. J Infect Dis. 1998;178(1):27–34. 10.1086/515586. 9652419

[12] Bavinger JC, Anthony CL, Lindeke-Myers AT, Risk factors for retinal detachment in acute retinal necrosis. Ophthalmol Retina. 2022;6(6):478–83. 10.1016/j.oret.2022.01.016. 35114414

[13] Zhao XY, Meng LH, Zhang WF, Wang DY, Chen YX. Retinal detachment after acute retinal necrosis and the efficacies of different interventions: a systematic review and meta-analysis. Retina. 2021;41(5):965–78. 10.1097/iae.0000000000002971. 32932382

[14] Ding X, Chang RT, Zhang X, Clinical spectrum and possible pathogenesis of progressive outer retinal necrosis. Br J Ophthalmol. 2024;109(1):107–12. 10.1136/bjo-2023-325113. 39237291

[15] Kashiwase M, Sata T, Yamauchi Y, Progressive outer retinal necrosis caused by herpes simplex virus type 1 in a patient with acquired immunodeficiency syndrome. Ophthalmology. 2000;107(4):790–4. 10.1016/s0161-6420(99)00143-8. 10768344

[16] Walton RC, Byrnes GA, Chan CC, Nussenblatt RB. Fluorescein angiography in the progressive outer retinal necrosis syndrome. Retina. 1996;16(5):393–8. 10.1097/00006982-199616050-00005. 8912965

[17] Gore DM, Gore SK, Visser L. Progressive outer retinal necrosis: outcomes in the intravitreal era. Arch Ophthalmol. 2012;130(6):700–6. 10.1001/archophthalmol.2011.2622. 22801826

[18] Culbertson WW, Blumenkranz MS, Haines H, Gass DM, Mitchell KB, Norton EW. The acute retinal necrosis syndrome. Part 2: histopathology and etiology. Ophthalmology. 1982;89(12):1317–25. 10.1016/s0161-6420(82)34638-2. 6298683

[19] Hojjatie SL, Shantha JG, O’Keefe GD, Cytopathology of Vitreous Specimens in Acute Retinal Necrosis. Ocul Immunol Inflamm Oct-Nov. 2022;30(7–8):1609–16. 10.1080/09273948.2021.1922926. 34242097 PMC8742848

[20] Greven CM, Ford J, Stanton C, Progressive outer retinal necrosis secondary to varicella zoster virus in acquired immune deficiency syndrome. Retina. 1995;15(1):14–20. 10.1097/00006982-199515010-00003. 7754241

[21] Butler NJ, Moradi A, Salek SS, Acute retinal necrosis: presenting characteristics and clinical outcomes in a cohort of polymerase chain reaction-positive patients. Am J Ophthalmol. 2017;179:179–89. 10.1016/j.ajo.2017.05.006. 28501392

[22] Ozdemir Yalcinsoy K, Cakar Ozdal P, Inanc Tekin M, Karatepe MS, Ozdamar Erol Y. Acute retinal necrosis: clinical features, management and outcomes. Int Ophthalmol. 2023;43(6):1987–94. 10.1007/s10792-022-02598-7. 36436167

[23] Schoenberger SD, Kim SJ, Thorne JE, Diagnosis and Treatment of Acute Retinal Necrosis: A Report by the American Academy of Ophthalmology. Ophthalmology Mar. 2017;124(3):382–92. 10.1016/j.ophtha.2016.11.007. 28094044

[24] Classification Criteria for Acute Retinal Necrosis Syndrome. Am J Ophthalmol Aug. 2021;228:237–44. 10.1016/j.ajo.2021.03.057. 33845012 PMC8675365

[25] Takase H, Okada AA, Goto H, Development and validation of new diagnostic criteria for acute retinal necrosis. Jpn J Ophthalmol Jan. 2015;59(1):14–20. 10.1007/s10384-014-0362-0. 25492579

[26] Lei B, Zhou M, Wang Z, Chang Q, Xu G, Jiang R. Ultra-wide-field fundus imaging of acute retinal necrosis: clinical characteristics and visual significance. Eye (Lond). 2020;34(5):864–72. 10.1038/s41433-019-0587-8. 31554945 PMC7182555

[27] Huynh TH, Johnson MW, Comer GM, Fish DN. Vitreous penetration of orally administered valacyclovir. Am J Ophthalmol. 2008;145(4):682–6. 10.1016/j.ajo.2007.11.016. 18226802

[28] Tibbetts MD, Shah CP, Young LH, Duker JS, Maguire JI, Morley MG. Treatment of acute retinal necrosis. Ophthalmology. 2010;117(4):818–24. 10.1016/j.ophtha.2009.09.001. 20079537

[29] Palay DA, Sternberg P Jr., Davis J, Decrease in the risk of bilateral acute retinal necrosis by acyclovir therapy. Am J Ophthalmol. 1991;112(3):250–5. 10.1016/s0002-9394(14)76725-x. 1882936

[30] Debiec MR, Lindeke-Myers AT, Shantha JG, Bergstrom CS, Hubbard GB 3rd, Yeh S. Outcomes of Combination Systemic and Intravitreal Antiviral Therapy for Acute Retinal Necrosis. Ophthalmol Retina Mar. 2021;5(3):292–300. 10.1016/j.oret.2020.07.012. 32683108 PMC8477308

[31] Wong R, Pavesio CE, Laidlaw DA, Williamson TH, Graham EM, Stanford MR. Acute retinal necrosis: the effects of intravitreal foscarnet and virus type on outcome. Ophthalmology. 2010;117(3):556–60. 10.1016/j.ophtha.2009.08.003. 20031221

[32] Yeh S, Suhler EB, Smith JR, Combination systemic and intravitreal antiviral therapy in the management of acute retinal necrosis syndrome. Ophthalmic Surg Lasers Imaging Retina Sep-Oct. 2014;45(5):399–407. 10.3928/23258160-20140908-02. 25215870

[33] Fan S, Lin D, Wu R, Wang Y. Efficacy of prophylactic laser retinopexy in acute retinal necrosis: a systematic review and meta-analysis. Int Ophthalmol. 2022;42(5):1651–60. 10.1007/s10792-021-02131-2. 35307785

[34] Anthony CL, Bavinger JC, Yeh S. Advances in the diagnosis and management of acute retinal necrosis. Ann Eye Sci. 2020. 10.21037/aes-2019-dmu-09. PMC7771653 33381683

[35] Shantha JG, Weissman HM, Debiec MR, Albini TA, Yeh S. Advances in the management of acute retinal necrosis. Int Ophthalmol Clin Summer. 2015;55(3):1–13. 10.1097/iio.0000000000000077. PMC4567584 26035758

[36] Kalogeropoulos D, Afshar F, Kalogeropoulos C, Vartholomatos G, Lotery AJ. Diagnostic and therapeutic challenges in acute retinal necrosis; an update. Eye (Lond). 2024;38(10):1816–26. 10.1038/s41433-024-03028-x. 38519714 PMC11226642

